# Antibody therapies for melanoma: New and emerging opportunities to activate immunity (Review)

**DOI:** 10.3892/or.2014.3275

**Published:** 2014-06-20

**Authors:** SADEK MALAS, MICAELA HARRASSER, KATIE E. LACY, SOPHIA N. KARAGIANNIS

**Affiliations:** St. John’s Institute of Dermatology, Division of Genetics and Molecular Medicine and NIHR Biomedical Research Centre at Guy’s and St. Thomas’ Hospitals, King’s College London, London SE1 9RT, UK

**Keywords:** antibodies, melanoma, immunotherapy, host immunity, immunosuppression, effector cells, cytotoxicity, checkpoint blockade

## Abstract

The interface between malignant melanoma and patient immunity has long been recognised and efforts to treat this most lethal form of skin cancer by activating immune responses with cytokine, vaccine and also antibody immunotherapies have demonstrated promise in limited subsets of patients. In the present study, we discuss different antibody immunotherapy approaches evaluated in the context of melanoma, each designed to act on distinct targets and to employ different mechanisms to restrict tumour growth and spread. Monoclonal antibodies recognising melanoma-associated antigens such as CSPG4/MCSP and targeting elements of tumour-associated vasculature (VEGF) have constituted long-standing translational approaches aimed at reducing melanoma growth and metastasis. Recent insights into mechanisms of immune regulation and tumour-immune cell interactions have helped to identify checkpoint molecules on immune (CTLA4, PD-1) and tumour (PD-L1) cells as promising therapeutic targets. Checkpoint blockade with antibodies to activate immune responses and perhaps to counteract melanoma-associated immunomodulatory mechanisms led to the first clinical breakthrough in the form of an anti-CTLA4 monoclonal antibody. Novel modalities to target key mechanisms of immune suppression and to redirect potent effector cell subsets against tumours are expected to improve clinical outcomes and to provide previously unexplored avenues for therapeutic interventions.

## 1. Clinical landscape

Malignant melanoma is responsible for ~75% of all skin cancer-related deaths with 250,000 new cases diagnosed yearly worldwide. The 5-year survival rate is ~95% for patients diagnosed with stage I disease and markedly falls to <10% for patients with metastatic stage IV disease (1). Until recently, available treatments were limited. The most commonly used chemotherapeutic agent to treat patients with advanced melanoma has for many years been dacarbazine, an alkylating agent licenced in 1975 to treat unresectable stage IV disease. Dacarbazine works by inducing cell cycle arrest and tumour cell apoptosis and treatment results in a response rate of ~15–20% and median response duration of ~4 months (2). Immunotherapeutic strategies in the form of the recombinant cytokines IL-2 and IFNα, although not universally applied in standard practice due to their side-effects, support the notion that activating immunity in cancer may have a positive clinical impact (3). The recent regulatory approval of two classes of agents has transformed clinical care: the pathway inhibitor small drugs vemurafenib, dabrafenib and trametinib; and a monoclonal antibody, known as ipilimumab, that targets the T cell checkpoint molecule CTLA4 to promote T cell activation (Table I) (4–7).

### Insights from the interplay with immune responses

Melanoma has for decades been considered an immunogenic tumour. The presence of specific immune cell subsets such as T lymphocyte infiltrates within melanoma lesions is thought to correlate with favourable patient outcomes (8). Clinical observations of partial or whole regressions of melanoma lesions, clinical evidence of spontaneous complete remissions, the increased prevalence of melanoma in immunocompromised patients and the mixed success of immune-based therapies dating back over many years, further support an important role for the tumour-immunity interface in melanoma.

Melanoma cells are thought to potentiate an inflammatory-based response constituting various cell subsets such as cytotoxic T cells (CTLs), dendritic cells (DCs), macrophages, neutrophils, mast cells, T and B lymphocytes and also an array of cytokines and antibodies within the tumour and systemically (9–11). However, local and systemic immunity often fails to stem cancer progression. It has been postulated that the immune system struggles to balance mounting a sufficient immune response against altered self-proteins expressed on tumours on the one hand, while aiming to avoid unwanted responses against self, on the other (12). Tumour-associated immunosuppressive components such as regulatory T cells (Tregs), myeloid-derived suppressor cells (MDSCs) and alternatively activated (M2d) macrophages, along with mediators such as IL-10, transforming growth factor (TGF-β) and vascular endothelial growth factor (VEGF) promote inflammation and immune suppression, diverting T and B cell responses and antibody expression in favour of inactive or suppressive subclasses such as IgG4 and preventing potentially cytotoxic T cells, DC and macrophages from launching potent antitumoural functions (13–16). Therefore, interactions between tumours and immunity are often tipped in favour of tumour growth.

The limited positive impact of standard chemotherapeutics on advanced disease survival and evidence that host immunity is capable of detecting the presence of tumours have long provided the motivation to design new therapy approaches, including those with the capacity to activate or redirect immune responses against melanoma.

### Cytokine therapies

Early efforts to harness immunity focused on recombinant cytokines IFNα2b and IL-2 (3,17). IL-2 was used on the basis that it could have antitumour effects by inducing expansion of tumour-specific T cell populations (18). However, treatment with high dose IL-2 for advanced disease resulted in an objective response rate of 5–27% and complete responses of up to 4% of patients in randomised controlled trials (19). High dose treatments have serious side-effects, such as hepatic and renal toxicities and high mortality rates (Table I) (20).

IFNα, a member of the type I-IFN family, is an antiviral and powerful pro-inflammatory cytokine produced by a wide variety of cells (including T cells, NK cells, macrophages). Shown to inhibit proliferation of B16 murine melanoma cells *in vivo* and *in vitro*, it is thought to have antitumoural effects via anti-angiogenic and pro-apoptotic functions and by enhancing tumour antigen presentation and potentiating CD4^+^ T cell-mediated tumour cell targeting (21,22). High doses of IFNα have been shown to prolong disease-free survival and, when used in the adjuvant setting, the agent could increase overall survival in high-risk patients (23), although treatment was not demonstrated to result in significant survival improvements when compared with chemotherapy in randomised trials. Although approved for clinical use and used in the adjuvant setting in Europe, INFα is not standard of care in the United Kingdom (24,25). Recent investigations have shown that a modified form of IFNα (pegylated IFNα2β) with a longer half-life *in vivo* has enhanced therapeutic efficacy and improved tolerability, leading to FDA approval as an adjuvant therapy in 2011 (Table I) (26).

### Vaccine approaches

Vaccination strategies have been investigated using peptides, proteins, cells, DNA and viral vaccines or various forms of modified cell therapies such as adoptive DC and T cell therapies; a DC therapy is available for the treatment of prostate cancer, offering hope for similar treatments in melanoma (27). Tumour cells used as immunogens, melanoma peptide and protein recombinant antigens or DNA viral vector vaccines have been designed to stimulate different components of the immune response and these efforts continue to date (28). A recent study showed induction of immunity against metastatic melanoma through vaccination with mature DCs loaded with melanoma antigens (MART-1, MAGE-3, gp100 and tyrosinase) processed through melanoma constitutive proteasomes for presentation by MHC class I to cognate T cells. Treatment enhanced Ag-specific T cell responses and reduced levels of circulating tumour cells in all patients (29). A phase I study of a murine gp100 DNA vaccine in malignant melanoma patients showed that the delivery of xenogeneic melanoma antigens (Tyr, gp100) can activate a specific CTL response to these proteins, with low associated toxicity and gp100-reactive T cell responses were reported in some patients, but without improving median survival (30). Although limited by their individual patient-specific nature, reliance on high expertise and high associated costs, adoptive cell strategies are now accelerated by the emergence of new technologies. To date, however, vaccines demonstrating clinical benefits have not reached clinical utility in melanoma.

## 2. Monoclonal antibodies

**Figure 1:**
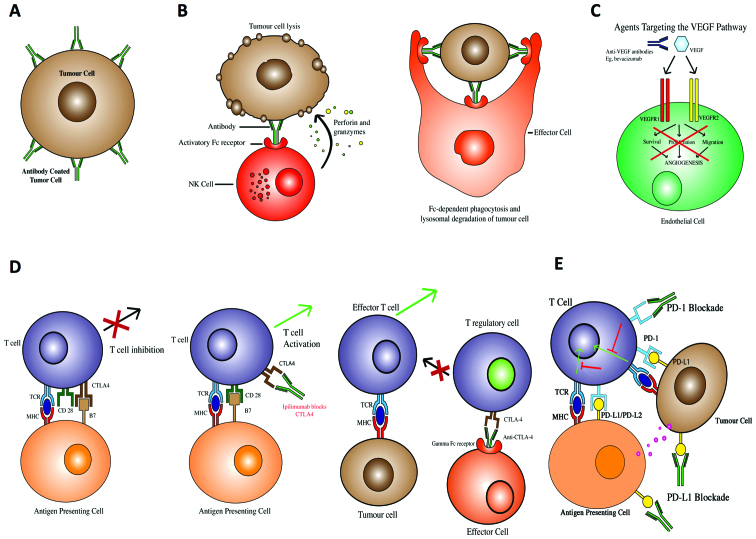
Possible mechanisms of action employed by monoclonal antibodies. (A) Direct effects by specific recognition of cell surface target antigens, triggering possible downstream signalling events that lead to target cell death. (B) Antibody-dependent cell-mediated cytotoxicity (ADCC) is based on antigen target-reactive antibodies coating tumour cells recognised by Fc receptors expressed on immune cells such as NK cells, macrophages and neutrophils; these lead to effector cell activation and tumour cell death (left). Antibody-coated tumour cells can also engage Fc receptors present on phagocytes leading to antibody-dependent cell-mediated phagocytosis (ADCP) (right) of the target cell. (C) Targeting VEGF prevents its association with its cell surface receptors (VEGFR1, VEGFR2) preventing downstream signals that lead to formation of tumour-associated vasculature. (D) Targeting the checkpoint molecule CTLA4; CTLA4 binds to B7 on antigen presenting cells (APC) and interferes with CD28-B7 complex co-stimulatory signals needed for MHC-antigen-TCR antigen presentation, thus inhibiting T cell activation. Antibodies such as ipilimumab block binding of CTLA4 to B7 on the surface of APCs; this allows CD28-B7 complex assembly and co-stimulation that restores T cell activation. Ipilimumab also activates Fc receptor-expressing effector cells against CTLA4-expressing Tregs, leading to their elimination via ADCC. (E) Targeting the PD-1:PD-L1 interactions with antibodies: PD-1 on the surface of antigen-educated T cells engages with PD-L1 expressed on melanoma cells and on other immune cells in tumours, leading to T cell anergy and/or deletion. PD-1:PD-L1 complex formation may play a role in tumour immune escape and antibodies targeting the PD-1/PD-L1 blockade may activate T cell responses against melanoma.

Antibody-based agents have been increasingly used as therapies for a wide range of human malignancies, including some solid tumour indications such as breast, colorectal and lung cancers (31). Antibodies can exert their antitumoural functions directly by specific recognition of cell surface antigen-expressing target cells, such as signalling proliferation arrest, inducing apoptosis, blocking cytokine receptor interactions to starve tumour cells of vital growth signals, or preventing tumour cell-extracellular matrix interactions to restrict migration and metastasis (Fig. 1A). Antibodies can also link target antigen expressing cells (such as tumour cells) with immune effector cells bearing Fc receptors, potentiating effector cell activation and target-neutralising functions (Fig. 1B) by engendering antibody-dependent effector cell-mediated cytotoxicity (ADCC), phagocytosis (ADCP) or complement activation (CDC). Antibodies can also be used as immunogens, to promote antigen presentation and initiate adaptive immune responses against cancer cells, or by targeting key elements of immune modulatory pathways to overcome effector cell anergy. Another function may entail targeting critical events in the tumour microenvironment, such as VEGFs to inhibit angiogenesis, restricting tumours of vital nutrient supply and/or escape of metastatic cells into the circulation (Fig. 1C).

Studies on the merits of antibody therapies for the treatment of melanoma date back a few decades, but the clinical utility of this modality has only recently been demonstrated with the approval of the first antibody in 2011. Renewed attention is now focused towards melanoma immunotherapies including monoclonal antibodies that can target key cancer pathways and activate immunity (32). Here, we discuss examples of different antibody therapeutic strategies studied for melanoma.

### Targeting cell surface antigens on tumour cells: CSPG4

A widely used approach for antibody therapies entails selective recognition of specific molecules such as proteins, sugar or lipid moieties, which are overexpressed or mutated on the surface of cancer cells. In the context of melanoma, a notable example of this strategy includes antibodies designed to recognise the tumour-associated antigen chondroitin sulphate proteoglycan 4 (CSPG4). Otherwise known as melanoma-associated chondroitin sulphate proteoglycan (MCSP) or human high molecular weight-melanoma associated antigen (HMW-MAA), CSPG4 is a 2322-residue membrane-bound protein overexpressed on 80–85% of melanoma lesions and also on a proportion of basal cell and breast carcinomas and several leukaemia types (33,34).

CSPG4 is known to play roles in the adhesion, spreading and migration of melanoma cells through activation of intracellular signalling cascades such as the focal adhesion kinase (FAK), PI3K/AKT, NFκB and MAPK/ERK 1/2 pathways, promoting sustained high levels of activatory signals required for malignant progression (35–37). In addition to melanoma cells, CSPG4 is expressed at high levels in tumour angiogenic vasculature, providing the potential for a targeted therapy to also restrict tumour blood supply (38). Overexpression in melanomas at different stages, restricted tissue distribution in normal tissues and a central role in cancer cell motility, metastasis and tissue invasion, render CSPG4 an attractive therapeutic target for applications, including monoclonal antibodies that may be used in adjuvant and in advanced disease settings.

Monoclonal antibodies and antibody fragments have been examined with the view to develop targeted therapies against CSPG4-expressing tumours. Antibodies conjugated to radioisotopes or toxins (purothionin, pseudomonas exotoxin A, methotrexate) could induce tumour cell clearance by antibody-targeted delivery of these moieties to melanoma cells. Another early approach aimed at overcoming immunological unresponsiveness to CSPG4 entailed designing anti-idiotypic antibodies to act as immunogens by mimicking tumour antigen epitopes known to be recognised by patient humoral responses. Early clinical trials reported the development of anti-CSPG4 antibodies associated with prolonged survival of melanoma patients who responded to antibody therapy (39–43). Host anti-CSPG4 antibodies were detected in ~60% of patients after treatment with an anti-idiotypic antibody in conjunction with an adjuvant (BCG) and those individuals who developed antibodies also showed longer median survival (42–44).

A humanised bi-specific BiTE antibody (bi-specific T-cell engaging), able to bind both CSPG4 and human CD3 to engage the T cell receptor (TCR) complex, was designed to redirect CD3^+^ T cells (especially CTL) against melanoma cells (45). This strategy aimed at triggering T cell activation in the absence of specific T cell clones or antigen presentation by APC. When peripheral blood mononuclear cells (PMBCs) from healthy donors co-cultured with melanoma cells were treated with MCSP-BiTE antibodies at different doses, PBMCs lysed CSPG4-expressing melanoma cells in an antigen-specific manner. Notably, similar cytotoxic activity was observed with T cells from melanoma patients, although the proportions of CD3^+^ T cells in patient blood were lower compared to those from healthy volunteers.

An anti-CSPG4 scFv antibody fragment fused with human TRAIL (TNF-related apoptosis-inducing ligand) was designed to deliver pro-apoptotic TRAIL-signalling activity when bound to CSPG4^+^ melanoma cells, while inhibiting antitumourigenic signalling via CSPG4 downstream signal blocking. Previous studies showed the inhibition of CSPG4^+^ melanoma cells *in vitro* and *in vivo* with this agent and reported that antitumoural activity was enhanced in the presence of the sigma receptor (σR) rimcazole, also known to have selective antitumoural activity (46,47). A more recent approach entails engineered T cells with a CSPG4-specific chimaeric antigen receptor also encoding the CD28 co-stimulatory endodomain. CAR-CSPG4-expressing T cells demonstrated antitumoural activities *in vitro* and in mouse models of melanoma, mesothelioma, breast and head and neck squamous cell carcinomas (48). Several years of promising findings with a variety of approaches based on CSPG4 as a tumour target, together with recent technical, translational and clinical developments in the field of antibody therapeutics for solid tumours, now provide a strong case for revisiting the concept of an antibody modality centred on this promising tumour antigen.

### Targeting tumour vasculature: VEGF

Malignant melanoma progression from the radial to the vertical growth phases has been associated with increased microvessel density and poorer outcomes for patients with high tumour-associated vasculature. Melanoma tumours feature increased production of pro-angiogenic factors such as the VEGF family molecules, VEGF-A involved in angiogenesis, VEGF-B associated with embryonic angiogenesis, VEGF-C with known roles in lymphangiogenesis, VEGF-D participating in lung bronchiole lymphatic vasculature, and VEGF-E encoded from a gene of viral origin with endothelial cell proliferative properties. Production of VEGFs, especially VEGF-A, VEGF-C and VEGF-D, and their receptors VEGFR-1, VEGFR-2 and VEGFR-3 trigger changes in endothelial cell proliferation, support formation of *de novo* blood vessels and vascular permeability at different tumour sites, to allow a higher degree of tumour cell survival and distal metastases. During tumour growth, VEGF-A, overexpressed in many solid tumours including melanoma, can stimulate a hypoxia-driven cascade of endothelial cell proliferation and migration leading to angiogenesis via recognition of the class III tyrosine kinase receptors (VEGFR-1 and 2) (49,50). Melanoma lymph node metastases involve higher expression and engagement of the VEGF-C/D and VEGFR-3 system.

The hypothesis that blocking VEGF-VEGFR interactions may be beneficial for patients led to the development of bevacizumab, a humanised monoclonal antibody recognising a high affinity epitope expressed on all VEGF-A isoforms. The antibody blocks VEGF binding to both receptors (Fig. 1C) (51,52) and has been approved by FDA for the first-line treatment of metastatic colorectal and breast cancers. A phase II study of this antibody as a first-line treatment for metastatic melanoma, in combination with the cytotoxic nitrosourea alkylating agent fotemustine, significantly reduced systemic levels of VEGF-A and VEGF-C, and also of the receptors VEGFR-1 and VEGFR-2. These findings suggested that this therapy contributes to the inhibition of angiogenesis and lymphangiogenesis, both of which are highly relevant in the context of melanoma progression (53). The mean time to progression was reported to be 8 months and overall survival was 20.5 months in previously untreated patients with advanced disease. Other trials, such as a phase II trial testing bevacizumab in combination with low dose IFN-α2b or standard chemotherapy, showed low activity and general minimal toxicity in addition to a stable disease state prolonged in some patients (54,55). The adjuvant avastin trial in high-risk melanoma (AVAST-M) sponsored by Cancer Research UK, a large phase III study for patients with stage IIB, IIC and III cutaneous melanoma following resection aimed to evaluate the capacity of bevacizumab to prevent disease recurrence in the adjuvant setting, when early angiogenesis may be prevented from assisting formation of early metastases. Results from the AVAST-M trial demonstrated good tolerability but noted no significant differences in the overall survival between treatment and observation groups (56). Other approaches may include antibodies directly targeting VEGF receptors (e.g. combinations of antibodies such as bevacizumab with chemotherapies or immunotherapies (57–59).

### Focus on T cell checkpoint cascades: CTLA4

The cytotoxic T-lymphocyte antigen-4, CTLA4, expressed on the surface of activated T lymphocytes, acts as an inhibitory molecule of T cell activation by competing with CD28 for binding to the co-stimulatory B7 family members on antigen-presenting cells (60,61). This leads to inhibition of TCR activity, reducing IL-2 gene transcription and T cell proliferation. CTLA4 acts as a negative regulator of T cell activation, contributing to antigen tolerance and limiting T cell mediated autoimmunity and homeostasis maintenance. Since tumour antigens are largely self-antigens, it was hypothesised that blocking CTLA4-B7 interactions to enhance T cell activation could help overcome tumour antigen tolerance and consequently potentiate enhanced antitumoural immune responses.

Ipilimumab, a human IgG1 antibody recognising CTLA4, interferes with CTLA4-B7 interactions on the surface of antigen presenting cells, permitting CD28-B7 complex formation. The antibody has been shown to operate by reducing CTLA4-induced T cell inhibitory functions, but also by targeting activatory Fc receptor-expressing effector cells against CTLA4-expressing Tregs, thereby blocking their immunosuppressive functions in tumours (Fig. 1D) (62–64). In early clinical studies, this agent showed adequate safety and an indication of efficacy, leading to subsequent trials at the National Cancer Institute (USA) that demonstrated sustained responses (>2 years) in a proportion of patients. Grade 3 or 4 adverse events such as colitis, rash and liver function abnormalities were observed in 19% of patients. A randomised, multi-institution, double blind, dose-ranging clinical study showed encouraging results with response rates as high as 11.1% and survival data as high as 30% (65–67). Combination studies of administration of ipilimumab with dacarbazine indicated increased response rates with combination treatment when compared with single treatment controls (68,69). A phase III trial was the first randomised study to show a survival benefit in patients with metastatic melanoma: ipilimumab (3 mg/kg dose) demonstrated median overall survival of 10 months and 10.1 months when combined with an HLA-A^*^0201-restricted gp100 vaccine peptide, while gp100 peptide treatment alone showed a median overall survival of 6.4 months (70).

Ipilimumab (at 3 mg/kg once every three weeks for four times) was the first antibody to be approved in 2011 by the FDA in the USA and soon afterwards in Europe for the treatment of patients with unresectable or metastatic melanoma (stage III and IV disease) (Table I) (71). This was a significant milestone as this was the first antibody to demonstrate significant survival benefits in patients with advanced melanoma (72). In clinical application, ~40% of patients experience immune-related adverse events (irAEs) through the universal activation of T cells, leading to tissue-specific inflammation and autoimmune-related side-effects, such as dermatitis, colitis and hepatitis. These side-effects can be managed with systemic steroid treatments without significant effects on benefit from the antibody therapy, but highlight the need for careful patient selection in addition to frequent monitoring. Comprehensive toxicity management algorithms have been developed to help manage patients, especially as most of the toxicities are reversible with early intervention. It is noteworthy that patients who experience autoimmune-related adverse events are more likely to benefit from treatment with ipilimumab, pointing to a fine balance between autoimmunity and the clearance of tumours (73).

Tremelimumab, a human IgG2 anti-CTLA4 monoclonal antibody, was also examined. Although it demonstrated durable objective tumour regressions in early clinical trials, it was less effective than ipilimumab, with lower response rates and median survival and a phase III study indicated no superior benefits relative to those observed with dacarbazine (74).

Although ipilimumab is the only antibody therapy to date that has shown significant impact on overall survival in advanced melanoma, ongoing late phase clinical trials are presently exploring anti-CTLA4 antibodies alone or in combination with other antibodies, immunotherapies and chemotherapeutic agents (Tables I and II).

### The PD-1/PD-L1 axis

Programmed death-1 (PD-1, CD279, or B7-H1), a member of the B7:CD28 group of cell surface molecules with homology to CTLA4, is an inhibitory cell surface protein expressed on the surface of mature, antigen-experienced T and B lymphocytes and myeloid cells following activation. Its ligand, PD-L1, is upregulated on the surface of antigen presenting cells in addition to epithelial cells and vascular endothelial cells upon antigenic stimulation with inflammatory signals such as IFNγ. PD-L1 has also been shown to be expressed on melanoma cells and other immune cells in tumours. Engagement of PD-1 to PD-L1 can inhibit T cell growth and cytokine secretion. PD-1 may play a critical role in tumour immune escape by engaging with PD-L1 and negatively regulating both cellular and humoral immune responses, permitting tumours to evade immune surveillance. PD-1 and PD-L1 expression is also linked to poor clinical prognosis. Therefore, blocking their interactions has been investigated as a possible immunotherapy strategy (Fig. 1E) (75–77).

A phase I clinical trial of the human IgG4 antibody nivolumab (MDX-1106) recognising PD-1 in melanoma (among other refractory cancers) has shown encouraging clinical activity and safety profiles (78). Nivolumab was also recently tested in further clinical studies, with reported median overall survival of 16.8 months, 62% 1-year and 43% 2-year survival rates. Grade 3 and 4 toxicities were found in only 5% of patients and clinical responses persisted even after cessation of therapy (79,80). A recent study showed that treatment with another anti-PD-1 humanised IgG4 antibody lambrolizumab in patients with advanced melanoma led to a tumour regression response rate of 38% (according to the response evaluation criteria in solid tumours, RECIST), with a durable effect and a median progression-free survival >7 months. Common, mostly low grade adverse effects observed during the treatment were fatigue, pruritus and rash (81).

Antibodies targeting PD-L1 found to be upregulated on tumour cells and on tumour-associated APCs triggered a high level of infiltrating T cells within tumour environments. A fully-human IgG4 antibody to PD-L1 that blocks association with PD-1 as well as with CD80 was tested in early clinical studies in different solid tumours including melanoma. Objective responses were observed in a proportion of patients with melanoma and durable responses were reported in a small proportion of patients (82). Any therapeutic effects of the PD-1/PD-L1 blockade continue to be examined in ongoing experimental and clinical studies.

### Depleting Tregs: anti-CD25

Tregs are considered a key suppressive cell subset which has high relevance in modulated antitumour responses. It has been suggested that Tregs may be partly responsible for the limited efficacy of adjuvant immunotherapies and vaccines (83). Tregs have been identified in primary melanomas, different metastatic sites and lymph nodes with melanoma cell metastases, and higher densities of Tregs correlate with thicker primary melanomas in the vertical growth phase (84,85). Increased numbers of skin resident Tregs are also observed in the skin of older people, indicating that increased age may entail less effective immune activation (86). Studies on tumour models suggest a link between Treg depletion and enhanced antitumour immunity. Targeting this cell subset has therefore been considered as a potential strategy to enhance the potency of immunotherapies. Daclizumab, a human anti-CD25 antibody was found to be successful in deleting circulating Tregs in patients with melanoma; however, depletion did not enhance the efficacy of a DC vaccine (87). An important limitation is that CD25 is not exclusively expressed by Tregs, but is also expressed on other activated T cell subsets, with potential harmful consequences associated with an anti-CD25 targeted therapy in weakening other crucial components of immunity.

## 3. Examples of combination strategies with antibodies

### Combination of anti-CTLA4 and anti-PD-1

Another approach is based on combining the blockade of both CTLA4 and PD-1 with antibodies. This strategy resulted in reduced Treg density in addition to enhanced effector T cell infiltration in tumour lesions in a mouse melanoma model (88). The concept was recently examined in a phase I clinical trial, administering nivolumab (IgG4 antibody to PD-1) and ipilimumab (IgG1 antibody to CTLA4) in patients with stage III or IV melanoma. Different regimens were used: concurrent (both mAbs, followed bynivolumab alone and subsequent combined treatments), while patients pre-treated with ipilimumab were administered nivolumab (sequenced). A 2.5 year follow-up showed 53% objective responses in the concurrent treatment group with marked tumour reductions (the majority showing a tumour regression of ≥80%) compared to 20% objective response (OR) rate for sequenced treatments. Over half of patients administered concurrent treatments showed grade 3 and 4 toxicities (89). Thus, important advantages of this combination therapy were response durability and the increased proportion of patients experiencing responses, compared to monotherapies; further late phase clinical trials are now examining this concept (Tables II and III).

### Combination of mAb and vaccines

Combining mAb and vaccines comprising long-peptides of known melanoma- or melanocyte-associated antigens, such as gp100 and the tumour-differentiation antigens tyrosinase-related proteins (TRP)-1 and TRP-2 has been studied. In a melanoma mouse model, the altered long-peptide gp10025–33 vaccine together with the TLR7 ligand imiquimod (to activate gp100-specific CTL responses), and the mAb TA99 (specific for the melanocyte protein TRP-1) were used in combination (90). This led to activation of tissue-resident FcγR^+^ immune cells (e.g. macrophages), creating a critical window of time that allowed development of peptide vaccine-induced T cell responses. Delayed tumour growth and long-term survival responses were observed in >50% of the treated animals.

### Combination of anti-GD2, agonist CD40 mAbs and CpG

A combination of two monoclonal antibodies and a TLR9 ligand was examined. One antibody targeted the tumour antigen disialoganglioside-GD2, a glycosphingolipid overexpressed in neuroblastoma and melanoma. This antibody was engineered with reduced complement-dependent toxicity and improved ADCC functions. The second antibody was agonistic to CD40, a member of the TNF-receptor superfamily expressed on several cells such as DCs and macrophages. By targeting CD40, the antibody promoted tumour cell apoptosis, activated DC and macrophage activation and maturation. The antibodies were administered with the TLR9 ligand class B CpG ODN 1826 recognised by B cells, DCs and NK cells to enhance innate cell responses. This combination immunotherapy was more efficacious than single therapies in a model of GD2-expressing B16 melanoma at minimum tumour burden (91). Specifically, peritoneal macrophages from mice treated with anti-CD40 antibody and CpG were able to inhibit tumour cell proliferation *in vitro* and suggested a potential role *in vivo* in an antigen-dependent manner. Moreover, the antitumour efficacy of anti-GD2 *in vivo* was increased by anti-CD40 plus CpG therapy combinations.

## 4. Signalling pathway inhibitors: novel non-immunological treatments with immunological relevance

A proportion (~60%) of melanomas harbour mutations in the serine-threonine kinase BRAF, resulting in constitutive activation of the RAS/RAF/MEK/ERK pathway and irregular cell proliferation. The most common mutations (90%) involve a valine instead of a glutamic acid in the amino acid position 600, termed V600E mutation (92). Vemurafenib is licenced for patients whose tumours express the mutant form of the BRAF gene. This treatment has markedly greater response rates, overall survival rates and progression-free survival than dacarbazine, but most patients develop resistance and experience disease relapse (93). The BRAF inhibitors vemurafenib and dabrafenib, both selectively recognising mutant forms of BRAF, are now approved for clinical use for advanced stage disease (94).

Individually, ipilimumab and BRAF inhibitors improve overall survival and each has different clinical profiles; BRAF inhibition can result in quick but mostly temporary clinical responses, with median progression usually <7 months, whereas ipilimumab has a slow onset and a low rate of objective, but patients who respond to treatment experience long-lasting responses. It has also emerged that BRAF-inhibitors may improve antigen presentation and have been associated with reduced immunomodulatory responses and activated tumour-reactive T cells while patients respond to therapy (95,96). Thus, suggestions to combine an oncogene directed therapy with immunotherapy triggered substantial interest (97). Ribas *et al* conducted a Phase I study aiming to evaluate the safety and the best administration schedule for patients with metastatic BRAF V600-mutant melanoma. After treatment with both agents at the full approved doses (starting with vemurafenib for one month, then ipilimumab and concurrent vemurafenib), a few patients developed toxic effects such as increased aminotransferase levels and hepatic adverse events, both asymptomatic and reversible through administration of glucocorticoids or temporary termination of the therapy. These toxicities resulted in study closure (98). However, ongoing clinical trials continue to examine different strategies based on this concept.

## 5. Conclusions

Melanoma has historically been considered an immunogenic malignancy and has for long been resistant to standard treatments. Antibodies have always constituted a promising modality; however, with the development of checkpoint blockade antibodies and the approval of the first antibody for clinical use, their capacity to transform the treatment of patients is now better appreciated. Momentum is now gathering in the search for agents with capacity to potentiate immune clearance of melanoma tumours and to achieve tangible survival benefits (99). Parallel to the success of antibody therapies, pathway inhibitor drugs are also making a positive impact on disease management, despite drug resistance developing in most patients within months of treatment.

Beyond clinical application of checkpoint blocking antibodies, limitations of associated high toxicities and palpable clinical benefits for only a proportion of patients, immunotherapy of melanoma with antibodies now commands fresh consideration. The next generation of antibodies may be designed to re-educate and direct potent immune cells specifically against cancer cells. If achieved, a new class of agents may realize the capabilities of this modality to harness and potentially combine affinity and specificity for targets with directing potent subsets of immune effector cells against specific pathways or factors linked to cancer. Targeting inhibitory receptors such as PD-1 and PD-L1, to target tissue-resident antigen-educated T cell responses is likely to yield clinical data in the near future, and promises to potentiate T cell activation with potentially fewer toxic side-effects. Focus has also centred on combinations of antibodies with targeted pathway inhibitor drugs, other antibodies, vaccines or radiotherapy.

Yet to be explored in melanoma are emerging developments in antibody engineering including: bi-specific antibodies, including those combining tumour and immunological targets such as CD3 to focus T cells against tumours; toxin- or cytokine-loaded antibodies; modified antibodies with engineered Fc regions to enhance activatory effector functions (100). Numerous studies by us and others point towards consideration of antibodies of classes such as IgA or IgE with tissue immune surveillance functions that may be well-suited to the phenotypes of tumour-resident effector cells (101–107). Future studies may also provide further insights into the pathways associated with immune cell activation and re-direction in patient circulation and tumour microenvironments (11,14,15,62,108). Recent studies suggest the presence of an inflammatory B cell infiltrate in melanoma and biased production of IgG4, an antibody subclass with inefficient effector activities and with capacity to block cytotoxic antibodies from attacking tumours (10,11,14,15,109,110). New understanding of immunological mechanisms of response and strategies to counteract immunosuppression in melanoma may lead to new therapeutic interventions, but may also help identify predictive biomarkers for patient stratification or for monitoring responses to treatments (111). Clinically meaningful advances in the treatment of this challenging disease may now be in sight.

## Figures and Tables

**Table I:** Food and drug administration-approved agents for the treatment of malignant melanoma.

Agent (brand name)	Year of approval	Specificity	Class	Mechanisms of action
Dacarbazine (DTIC-Dome^®^)	1975	Non-specific	Chemotherapy	Alkylating agent leading to DNA damage, inducing cell cycle arrest and tumour cell apoptosis
IFNα2b (INTRON^®^A)	1995	IFNα receptor 1 and 2	Immunotherapy (cytokine)	Multifunctioning immunoactivatory cytokine enhances antitumoural response, anti-angiogenic, anti-proliferative and pro-apoptotic properties
High dose IL-2 (Aldesleukin, Proleukin^®^)	1998	IL-2 receptor expressed on lymphocytes	Immunotherapy (cytokine)	Immune activating, increases activation and proliferation of immune cells (e.g. T, NK, B cells)
Pegylated IFNα2b (PEG INTRON^®^A)	2011	IFNα receptor 1 and 2	Immunotherapy (cytokine)	Modified (pegylated) form of IFNα2b with increased half-life and enhanced therapeutic efficacy
Ipilimumab (Yervoy^®^)	2011	CTLA4 expressed on T cells	Immunotherapy (mAb)	Humanised mAb targeting the inhibitory receptor CTLA4 activates immune system enhancing T cell activation and targeting CTLA4-expressing Tregs
Verumafenib (Zelboraf^®^)	2011	BRAF V600E, mutated form of BRAF	Small molecule inhibitor	Blocks mitogen-activated protein kinase pathway reducing protein proliferation of melanoma cells carrying mutation
Dabrafenib (Tafinlar^®^)	2013	BRAF V600E mutated form of BRAF protein	Small molecule inhibitor	Blocks mitogen-activated protein kinase pathway reducing proliferation of melanoma cells carrying mutation
Trametinib (Mekinist^®^)	2013	BRAF V600E or V600K mutated forms of BRAF protein	Small molecule inhibitor	Blocks mitogen-activated protein kinase pathway reducing proliferation of melanoma cells carrying mutation

mAb, monoclonal antibody; IFN, interferon; IL, interleukin.

**Table II:** USA-registered phase III clinical trials of antibody therapies for melanoma.^a^

Drug/intervention	Drug type	Sequence of drug administration	Stage/cancer type	Identifier
Ipilimumab (i) vs. MD-1379 (ii) vs. Ipilimumab (i) + MD-1379 (ii)	Anti-CTLA4 monoclonal antibody (i) melanoma peptide vaccine (ii)	IV ipilimumab every 3 weeks for 4 doses MDX-1379 2 ml (2 subcutaneous injections of 2 ml each, 1 to each thigh), every 3 weeks for 4 doses	Unresectable or metastatic melanoma	NCT00094653
Ipilimumab (i) vs. recombinant IFNα2b (ii)	Anti-CTLA4 monoclonal antibody (i) IFNα2b (ii)	High dose ipililmumab IV over 90 min every 21 days for 4 courses. Then maintenance high-dose ipilimumab IV over 90 min every 90 days for a maximum of 4 courses High dose recombinant IFNα2b IV on days 1–5, 8–12, 15–19 and 22–26 then maintenance high dose on days 1,3 and 5	Resected high-risk melanoma	NCT01274338
CP-675, 206 (i) vs. Dacarbazine (ii) or Tremozolomide (iii)	Anti-CTLA4 human monoclonal antibody (i) alkylating chemotherapy agent (ii & iii)	CP-675, 206 15 mg/kg IV Q 90 days × 4 Dacarbazine 1,000 mg/kg IV Q 90 days × 4 Temozolomide 200 mg/m^2^ orally on days 1–5 every 28 days × 12	Advanced melanoma	NCT00257205
Ipilimumab (i) vs. placebo	Anti-CTLA4 monoclonal antibody (ii) placebo (ii)	IV ipilimumab every 21 days for 4 doses, then starting from week 24 every 12 weeks until week 156 or progression IV placebo 4 × every 21 days then starting from week 24 every 12 weeks until week 156 or progression	High-risk melanoma	NCT00636168
Nivolumab (i) vs. Nivolumab (i) + Ipilimumab (ii) vs. Ipilimumab (ii)	Anti-PD-1 monoclonal antibody (i) anti-CTLA4 monoclonal antibody (ii)	IV nivolumab every 2 weeks IV nivolumab with IV ipilimumab every 3 weeks for 4 doses then nivolumab IV every 2 weeks. IV ipilimumab every 3 weeks for a total of 4 doses	Untreated advanced melanoma	NCT01844505

a Source, www.clinicaltrials.gov.

IFNα2b, interferon α2b.

**Table III:** European- and UK-registered phase III clinical trials of antibody therapies for melanoma.^a^

Drug/intervention	Drug type	Stage/cancer type	Identifier
MK-3475 (i) vs. Ipilimumab (ii)	Anti-PD-1 monoclonal antibody (i) anti-CTLA4 monoclonal antibody (ii)	Advanced melanoma	2012-004907-10 (EU)
Response and/or toxicity vs. Ipilimumab (i)	Anti-CTLA4 monoclonal antibody (i)	Unresectable stage III or IV malignant melanoma	2005-002126-64 (EU)
Nivolumab (i) vs. investigator’s choice	Anti-PD-1 monoclonal antibody (i)	Unresectable or metastatic melanoma progressing post anti-CTLA4 therapy	13396 (UKCRN ID) (UK)
Nivolumab (i) vs. Ipilimumab (ii) vs. Nivolumab (i) + Ipilimumab (ii)	Anti-PD-1 monoclonal antibody (i) anti-CTLA4 monoclonal antibody (ii)	Unresectable or metastatic melanoma	14725 (UKCRN ID) (UK)

a Source, https://www.clinicaltrialsregister.eu; http://public.ukcrn.org.uk/search/.
